# Prolonged Toxic Encephalopathy following Accidental 4-Aminopyridine Overdose

**DOI:** 10.1155/2014/237064

**Published:** 2014-04-17

**Authors:** Maria Ballesta Méndez, Vincent van Pesch, Arnaud Capron, Philippe Hantson

**Affiliations:** ^1^Department of Intensive Care, Cliniques St-Luc, Université Catholique de Louvain, Avenue Hippocrate 10, 1200 Brussels, Belgium; ^2^Department of Neurology, Cliniques St-Luc, Université Catholique de Louvain, 1200 Brussels, Belgium; ^3^Louvain Centre for Toxicology and Applied Pharmacology, Université Catholique de Louvain, 1200 Brussels, Belgium

## Abstract

*Background*. 4-Aminopyridine (4-AP) is a drug that is used to improve motor fatigue in patients suffering from multiple sclerosis (MS). Medication error can occur, as commercial preparation may not be available in some countries. *Case Presentation*. A 58-year-old woman with progressive MS presented with status epilepticus. She was receiving 4-AP for more than 3 years. The symptoms started soon after the ingestion of a single pill that was supposed to contain 10 mg 4-AP, but further investigations revealed that each pill had been inadvertently prepared with an 100 mg 4-AP concentration. The patient was admitted to the intensive care unit (ICU) for appropriate management (orotracheal intubation, sedation, and antiepileptic drugs). The first electroencephalogram (EEG) showed abundant irregular spike-waves on the left central regions. Neurological condition gradually improved from day 7, while the EEG did not reveal any more electrical seizures but was still consistent with toxic encephalopathy. The patient stayed in the ICU until day 13. At discharge from the rehabilitation ward (2.5 months later), the patient had not yet recovered her previous cognitive and functional condition. *Conclusion*. A single 100 mg 4-AP accidental overdose may cause serious immediate complications, with a slow and incomplete neurological recovery.

## 1. Introduction

4-Aminopyridine (4-AP) is indicated for the improvement of walking speed and motor fatigue in multiple sclerosis (MS). An overdose in 4-AP can produce detrimental side effects. In the majority of cases, overdose is accidental and related to an error in the compounding of the drug [1, 2]. Due to the fact that no commercial preparation for 4-AP is available in Belgium, the medication must be compounded by the pharmacist upon medical prescription. We report a case of sustained encephalopathy following status epilepticus induced by 4-AP overdose.

## 2. Case Presentation

A 58-year-old woman with a medical history of secondary progressive MS was admitted to the emergency department (ED) for convulsive status epilepticus. Before admission, the Kurtzke Expanded Disability Status Score (EDSS) was 6.5/10. The patient was treated by 4-AP (10 mg orally, bid) for more than 3 years, without previous adverse effects. In addition, she was also receiving baclofen (10 mg bid), levothyroxine 50 *μ*g/d, citalopram 20 mg/d, and interferon-beta1b (3 subcutaneous injections per week).

**Figure 1:**
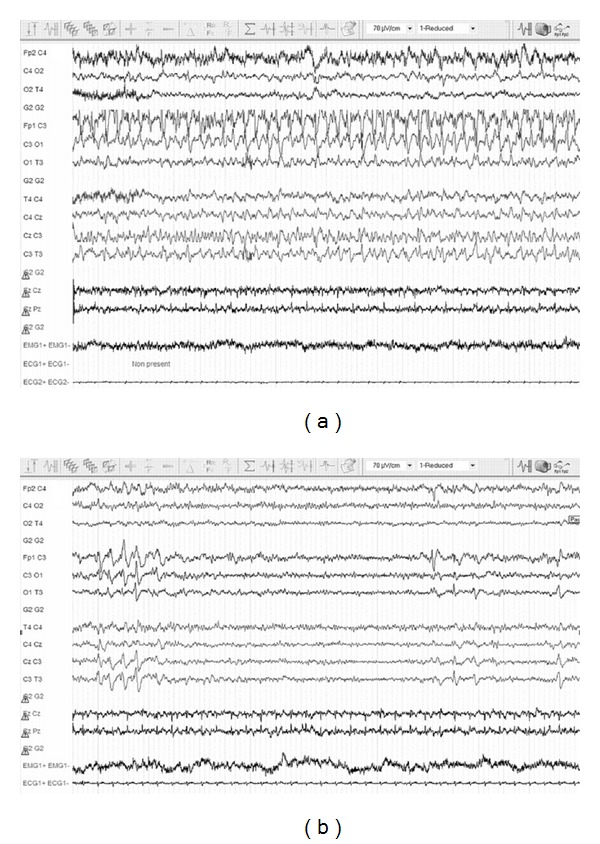
EEG recordings performed upon admission of the patient to the ICU (a) and at day 8 (b). (a) EEG traces show continuous epileptiform spikes and spike-waves at 2 Hz on the left frontocentral regions on a generalized slowed background intermixed with medicamentous beta waves. (b) EEG shows irregular short bursts of slow spikes and delta waves on the left frontocentrotemporal regions. Background activity at day 8 still shows diffuse beta waves, of medicamentous origin.

A few minutes after the ingestion of a single pill of 4-AP, she started to complain from severe abdominal pain. The pill came from a new box of a pharmacy preparation that was supposed to contain 10 mg of 4-aminopyridine per pill. Less than one hour after the ingestion, she developed rigidity, ocular revulsion, alteration of consciousness, and finally generalized tonic-clonic seizures. The first medical rescue team administered intravenous diazepam before hospital admission. On arrival in the ED, the patient had a Glasgow Coma Scale (GCS) score of 9 (E1V4M4). After recurrence of generalized tonic-clonic seizures, she received 2 mg lorazepam and 2 mg midalozam as intravenous bolus and a loading dose of 1200 mg valproic acid intravenously. Orotracheal intubation was required for mechanical ventilation. Sedation was obtained by continuous infusion of midazolam (5 mg/h) and propofol (100 mg/h). In the intensive care unit (ICU), after the regression of the effects of the neuromuscular blocking agent used for intubation, no hypertonia was observed. However, facial myoclonus rapidly reappeared. Antiepileptic therapy included a continuous intravenous infusion of valproic acid (1 mg/kg/h) together with levetiracetam (750 mg orally, bid). This resulted in the complete disappearance of any myoclonic or epileptic activity. Routine laboratory investigations were not relevant. Toxicological screening performed after ICU admission failed to reveal the presence of other substances than the patient's usual medications; there was no evidence for baclofen overdose. Brain magnetic resonance imaging (MRI) was similar to that obtained a few months before and confirmed the stability of the lesions related to MS. Double inversion recovery sequence imaging demonstrated the presence of intracortical demyelinating lesions in the insula and left parietal cortex. The patient was monitored in the ICU by continuous electroencephalographic (EEG) recording. The first EEG recording, performed upon admission to the ICU 12 hours after initial symptoms, showed generalized slowing at 6-7 cycles per second with abundant irregular spike- and polyspike-waves on the left frontocentral regions (Figure 1(a)). The epileptiform waves diffused at times to the right frontocentral regions and occasionally became rhythmic during 10 second bursts. On day 3, EEG monitoring showed a discontinuous pattern with bursts of electrical activity characterized by left frontocentral slow spikes at 1-2 Hz on a diffusely slowed background. As the sedation was reduced, EEG became gradually continuous with diffusely slow background activity at 6-7 cycles per second but still showed bursts of delta waves intermixed with slow spikes on the left frontocentral regions (Figure 1(b)).

Sedative drugs (midazolam 6 mg/h and propofol 200 mg/h) were maintained for 5 days for prevention of recurrent seizures. From day 1 to day 6, the GCS never exceeded 7 (E2V1M4). Starting from day 7, the patient was able to open the eyes spontaneously, but there was no interaction with the environment and no response to verbal command. On day 8, the patient was able to move spontaneously the four limbs. On day 11, she had a clear motor response (M6) to verbal command. Extubation was possible on day 12. She presented a painful dysphonia due to right vocal cord paresis. She had difficulties in understanding complex commands and remained confused. The patient developed left basal pneumonia as a late complication in the ICU. She was transferred to the neurology department on day 13. In addition to the altered cognitive state, the patient's neurological examination showed marked left predominant spastic paraparesis. Walking was impossible. The patient presented urinary retention and dysphagia. Cognitive testing revealed memory, executive, language, attentional, and visual-spatial difficulties. The EDSS was 8.5/10. EEG performed on day 22 showed a restored reactive structured background rhythm at 8.5–9.5 cycles per second, with predominant delta waves on the left frontal region, rarely intermixed with isolated slow spikes. On day 34, she was admitted to the rehabilitation ward, still confused and bedridden. She benefited from intensive physiotherapy, speech therapy, bladder training, and cognitive rehabilitation during 2.5 months. At discharge, she was able to walk a few meters with bilateral aid but needed partial aid with transfers. She could also swallow liquids and solid food. Her cognitive status and bladder function were partially improved. Her EDSS was 7/10.

The box containing the remaining pills was brought by the family to the hospital for toxicological analysis. A pill was analyzed by high performance liquid chromatography (HPLC) coupled to ultraviolet (UV) detection and was compared to a standard pill of 10 mg of 4-AP prepared by the hospital pharmacy. The 4-AP content of the pill that was ingested by the patient was at least 8 times greater (>80 mg) than the standard. The pharmacist admitted that each pill was erroneously prepared with a concentration of 100 mg.

## 3. Discussion

The exact pathophysiology of 4-AP toxicity remains to be elucidated. 4-AP is a potassium channel-blocking drug. The prolongation of the action potential may facilitate calcium entry into the cell; the increased influx of calcium is thought to enhance neurotransmission by releasing acetylcholine. Accidental 4-AP overdose may result in serious neurological events ranging from dystonia to altered consciousness and seizures [3, 4]. In a case series published by Burton et al. about three patients (46 to 54 years of age) who had ingested the first dose of a 4-AP preparation containing erroneously between 90.1 mg and 125.6 mg of 4-AP, unusual sensory and behavioral symptoms progressed within minutes to hours to status epilepticus [1]. In one patient only, the EEG revealed focal spike and wave activity. The patients required aggressive ICU treatment with intubation, benzodiazepines, and phenytoin. They were discharged with significant residual neurologic disability. However, seizures have to be clearly separated from other abnormal movements. In a recent review of the literature following their own case presentation, King et al. analyzed 19 cases of 4-AP toxicity and found that 5 patients presented isolated seizure and 6 status epilepticus. Intubation was performed not only in these 11 patients but also in 3 patients who did not experience seizures [3]. Benzodiazepines were administered to the majority of the patients as well as anticonvulsants (phenytoin or valproic acid) in reaction to seizures. However, some patients may present with dystonic choreoathetoid movements and altered consciousness [5–7]. The motor symptoms are most likely caused not by epileptogenic activity in the brain but by neuromuscular junction hyperactivity. Dysfunction of the basal ganglia, due to an altered acetylcholine release induced by 4-aminopyridine, could explain the abnormal involuntary, choreathetoid movements in the patients. This condition may be partly responsive to benzodiazepines but is not associated with epileptic changes on the EEG. The interpretation of the EEG may therefore be careful, with a distinction between nonconvulsive status epilepticus and toxic encephalopathy with specific electroencephalographic changes. The risk for the patient, if the interpretation of the EEG data is not correct, is that excessive pharmacological treatment could result in prolonged sedation, mechanical ventilation, and ICU stay. Our patient presented initial status epilepticus confirmed by initial EEG findings. From day 3 onwards, no further electrical seizures were recorded. EEG was consistent with a prolonged toxic encephalopathy, as also observed in other situations of drug overdose. For example, in MS patients treated by baclofen, overdose may also result in altered consciousness with epileptic-like EEG changes which do not require specific antiepileptic therapy [8]. In other case reports, 4-AP overdose has been shown to cause prolonged toxic encephalopathy without initial seizures. This cannot be explained by the kinetics of oral 4-AP. The determination of 4-AP blood concentration is not routinely available in most laboratories. It would be helpful in confirming exposure, but not for the assessment of acute neurotoxicity as there is an important overlap of 4-AP concentrations causing seizure or not. The drug is rapidly absorbed and is mainly excreted in the urine in an unchanged form (90.3%), with the role of hepatic metabolism being minimal. The serum terminal half-life of the molecule is between 5–6.5 hours and 24 hours. In contrast, the duration of toxic encephalopathy may be several days or even weeks. The neurologic recovery is not complete in all of the patients reported in the literature, as well as in our case [3]. Some patients suffered from a greater and chronic neurologic disability after the overdose episode; short or long-term memory loss is also a possible delayed complication [9]. The clinical course is not influenced by any specific treatment.

In conclusion, for the intensive care physicians, it is important to be aware of the clinical and electrophysiological specificities relating to 4-AP overdose, in order to avoid unnecessary treatments for nonconvulsive status epilepticus, together with prolonged sedation, mechanical ventilation and ICU stay.
